# Quest for breathing: proliferation of alveolar type 1 cells

**DOI:** 10.1007/s00418-022-02073-5

**Published:** 2022-01-20

**Authors:** Leszek Satora, Tomasz Gawlikowski, Adam Tański, Krzysztof Formicki

**Affiliations:** 1 Department of Hydrobiology, Ichthyology and Biotechnology of Reproduction, West Pomeranian University of Technology in Szczecin, ul. Kazimierza Królewicza 4, 71-550 Szczecin, Poland; 2 Department of Pharmacology, Clinical Pharmacology and Clinical Toxicology, Andrzej Frycz Modrzewski Krakow University, ul. G. Herlinga-Grudzińskiego 1, 30-705 Kraków, Poland

**Keywords:** Exaptation, Gut-breathing fish, Proliferation of alveolar type 1 cells

## Abstract

There is much evidence that the vertebrate lung originated from a progenitor structure which was present in bony fish. However, critical basic elements for the evolution of breathing in tetrapods, such as the central rhythm generator sensitive to CO_2_/pH and the pulmonary surfactant, were present in the lungless primitive vertebrate. This suggests that the evolution of air breathing in all vertebrates may have evolved through exaptations. It appears that the capability for proliferation of alveolar type 1 (AT1) cells is the “critical factor” which rendered possible the most radical subsequent innovation—the possibility of air breathing. “Epithelial remodeling,” which consists in proliferation of alveolar cells—the structural basis for gas diffusion—observed in the alimentary tract of the gut-breathing fishes (GBF) has great potential for application in biomedical research. Such a process probably led to the gradual evolutionary development of lungs in terrestrial vertebrates. Research on the cellular and molecular mechanisms controlling proliferation of squamous epithelial cells in the GBF should contribute to explaining the regeneration-associated phenomena that occur in mammal lungs, and especially to the understanding of signal pathways which govern the process.

## Introduction

The organs used for air breathing in vertebrates are very diverse (Duncker 2014). In spite of the differences in their structure and ventilation mechanisms, however, the respiratory organs always have some typical modifications: simple squamous epithelium and the distribution of numerous capillary vessels among the epithelial cells. The two characteristics must co-occur. Such an adaptation causes a significant reduction in the thickness of the air–blood barrier, allowing for gas diffusion. The adaptation is also observed in air-breathing fishes which use their stomach as an accessory respiratory organ (Satora 1998; Podkowa and Goniakowska-Witalińska 2003; Cruz et al. 2009; Cruz and Fernandes 2016). The lungs are the main organ of the respiratory system in mammals. They possess a unique architecture: millions of alveoli. Each of them is lined mainly by thin squamous epithelial cells. The apical parts of epithelial cells are strongly attenuated and form an exceedingly thin, continuous layer covering capillary blood vessels located between the bodies of epithelial cells; such structures are primarily sites for gaseous exchange (Liem 1988; Ciechanowicz 2019). Although mammalian lungs are slow-turnover organs that are highly quiescent at steady state, they have the ability to repair epithelial damage (Liem 1988; Ciechanowicz 2019).

Lung diseases are among the most common medical conditions all over the world. Moreover, chronic obstructive pulmonary disease (COPD) and lower respiratory infections are associated with high morbidity and mortality. Currently, they are ranked by the World Health Organization (WHO) as the third and fourth leading cause of death worldwide, respectively (WHO 2020). Both chronic and acute respiratory diseases affect the interstitium, for example sarcoidosis, idiopathic pulmonary fibrosis, autoimmune diseases, pneumonia, pulmonary edema (Kaku et al. 2020; Meyer et al. 2021). Parenchymal diseases are characterized by progressive remodeling of lung parenchyma combined with destruction and fibrosis of alveoli and, consequently, progressive respiratory distress. With the developing inflammatory reaction, the pneumocytes desquamate, which is accompanied by production of hyaline membranes. The altered alveoli cease to fulfill their primary role in gas exchange (Sims et al. 2005).

## Background

In mammals, gas exchange takes place in lung alveoli, which ensures a large surface area for diffusion of oxygen and carbon dioxide. The respiratory epithelium, which is the main component of the alveolar wall, contains two main types of cells (Liem 1988; Desai et al. 2014; Ciechanowicz 2019; Parekh et al. 2020). Alveolar type 1 (AT1) cells, which maximize surface area while minimizing the gas–blood barrier, occupy almost 95% of the lung surface and form a thin, continuous lining in the alveolar wall. AT1 cells supply a short diffusion pathway for gas exchange—creating a gas–blood barrier of about 0.2–2.5 µm (Liem 1988; Miettinen et al. 1997; Desai et al. 2014). Alveolar type 2 (AT2) cells (cuboid cells) are almost twice as abundant as AT1 cells (Ciechanowicz 2019), but they occupy only 7–10% of the lung surface and express high levels of surfactant protein C (Ciechanowicz 2019; Parekh et al. 2020). The surfactant ensures low surface tension and contributes to the elastic properties of the lungs (Bensch et al. 1964; Clements et al. 1970; Pattle 1976; Sullivan et al. 1998; Hawgood et al. 1998). In mammals, AT1 cells have lost their capacity for proliferation by cell division and, when damaged, they are replaced by AT2 cells (Liem 1988; Desai et al. 2014; Zacharias et al. 2018; Ciechanowicz 2019; Parekh et al. 2020). In fully developed lungs, the microenvironment regulates proliferation and differentiation potential of populations of multipotent endogenous stem cells located in niches (Ciechanowicz 2019). Furthermore, it is proposed that AT1 cells are completely differentiated, since there is little evidence to indicate that they can divide, whereas AT2 cells are regarded as bifunctional alveolar progenitor lung stem cells, which can differentiate into AT1 cells (Desai et al. 2014; Zacharias et al. 2018; Parekh et al. 2020). These functions are regulated by the bone morphogenetic protein (BMP) signaling pathway. During this process, BMP4 prevents proliferation of AT2 cells and promotes differentiation; its antagonists, such as Noggin, promote proliferation (Parekh et al. 2020). Other factors and signaling pathways are implicated in the self-renewal of AT2 cells after distal lung injury. In this case, stromal cell-derived factor 1 (SDF1) activates yes-associated protein (YAP), which leads to the production of growth factors, such as epithelial growth factor (EGF), and paracrine signals released by macrophages. However, following injury, AT2 cells possess limited proliferative ability. Further subclassification of AT2 cells, and ascertaining their role in lung regeneration processes, is still necessary (Desai et al. 2014; Zacharias et al. 2018; Parekh et al. 2020). However, without recognizing the mechanisms which control human lung development, the precise identity and function of human lung stem and progenitor cell types, and the genetic and epigenetic control of human lung fate, progress toward the development of strategies for lung regeneration following injury is impossible (Desai et al. 2014; Ciechanowicz 2019; Parekh et al. 2020).

## Research models

Studies on rodents—a group characterized by large disparities in the size, structure, cellular composition and physiology of their airways compared to humans—impose limitations on the use of this model as a preclinical animal model system (Parekh et al. 2020). On the other hand, differentiation of AT2–AT1 cells in 3D organoid culture in research on cell lineages is still the main challenge (Parekh et al. 2020). For example, freshly isolated cells of human alveoli quickly lose their differentiation status during culture, and this leads to failure to detect types of cells in vivo (Sims et al. 2005). Understanding the cellular and molecular mechanisms which control the development of the gas exchange surface and differentiation of the lungs is crucial for understanding the pathogenesis of acute and chronic lung diseases. This pertains especially to regeneration after exposure to damaging factors. Unfortunately, for obvious reasons, there is no direct physiological evidence, and the lung evolution can only be studied in extant species, followed by extrapolations (Randal et al. 1981). In this situation it seems crucial to find an adequate model to observe the initial stages of lung formation in terrestrial vertebrates (Satora et al. 2020b).

## From water to land

In the almost four billion years since life on earth emerged, evolution has generated a number of marvelous metamorphoses. One of the most spectacular changes is that which produced terrestrial creatures bearing limbs, fingers and toes from water-bound fish with fins. The replacement of fins with limbs was a crucial step in this transformation, but was by no means the only crucial step (Clack 2005). Land is a radically different medium from water, and to conquer this medium, tetrapods had to evolve novel ways to breathe and become equipped with a respiratory organ for air breathing. Most accounts of vertebrate evolution describe early air-breathing fishes, and stress the importance of aerial respiration in the origin of the tetrapods (Randal et al. 1981; Graham 1997). Nevertheless, the focus of these treatments usually shifts to the tetrapods themselves and the changes occurring in the phyletic progression from amphibians to mammals. Such accounts rarely consider the evolution of fishes beyond the Paleozoic and as a result succeed, more often than not, in conveying the impression that both fish evolution and the importance of air breathing to fishes ended with the appearance of amphibians. Similarly, comparative surveys of air-breathing fish respiratory adaptation have not considered the phyletic histories of fish, and thus most often treat both the primitive and modern air-breathing fishes similarly, as inferior grades of the mammalian specialization, evolutionary curiosities, or both (Randal et al. 1981; Graham 1997). Air breathing has persisted throughout the evolutionary history of the fishes and has played a fundamental role in the evolution of this group (Graham 1997; Icardo 2018). In general, air-breathing organs sequester a bubble of air out of contact with the water but in contact with a thin epithelium through which O_2_ diffuses into the blood. In contrast to water breathing, ventilation of the air-breathing organ is periodic (Kramer and Braun 1983).

## Exaptation

Exaptation is defined as existing structures that now enhance fitness but were not produced by natural (or sexual) selection for their current role (Gould and Vrba 1982; Tattersall 2009). There is much evidence to indicate that the vertebrate lung originated from a progenitor structure present in bony fish (Randall et al. 1981; Graham 1997; Nelson and Dehn 2011; Hoffman et al. 2016), but crucial structures for the evolution of air breathing were present in the vertebrate ancestors (lungless) prior to the evolution of the lung (Sullivan et al. 1998; Hoffman et al. 2016). In 2016, Hoffman and co-authors proposed a completely novel hypothesis, namely that the evolution of air breathing in all vertebrates occurred through exaptations (Gould and Vrba 1982; Tattersall 2009; 2014) derived from critical basic elements (Hoffman et al. 2016). One of them is the central rhythm generator sensitive to CO_2_/pH present in lamprey—a lungless vertebrate (Hoffman et al. 2016).

Additionally, Sullivan and co-authors point out that the evolution of air breathing must have been preceded by evolution of the surfactant system—evolved initially in the gut and subsequently utilized and modified in the lung (Sullivan et al. 1998). Pulmonary surfactant (mixture of lipids and proteins) is present in all air-breathing vertebrates, synthesized in the endoplasmic reticulum of cuboid alveolar cells (Bensch et al. 1964; Clements et al. 1970; Pattle 1976; Haagsman and van Golde 1991) and stored in dense multilayered structures called lamellar bodies (Bensch et al. 1964; Chevalier and Collet 1972). The surfactant forms a thin, amorphous alveolar lining, spreading over all the cells in contact with air (Bensch et al. 1964; Clements et al. 1970; Pattle 1976; Haagsman and van Golde 1991; Satora 1998). The surfactant reduces surface tension at the air–liquid interface and protects the cells against drying and the toxic effects of oxygen (Clements et al. 1970; Pattle 1976; Smits et al. 1994; Sullivan et al. 1998; Satora 1998). The study of surfactant protein A (SP-A) in members of all the major vertebrate groups implies that the surfactant had a single evolutionary origin in the vertebrates (Sullivan et al. 1998).

However, the presence of AT1 epithelial cells is indispensable for gas diffusion. The appearance of such epithelium, combined with the capability for proliferation, in the alimentary tract is the next critical element for the evolution of breathing in Tetrapoda. In addition, the ability of these cells to proliferate seems to be a “critical factor” of the practical breakthrough in the evolution of lung. The environmental factor—hypoxia—has turned out to be the main driving force of such changes leading to the origin of the lung (Randal et al. 1981; Graham 1997; Nelson 2014). According to Tattersall, exaptations, combined with “critical factors,” constitute a powerful evolutionary mechanism, and they are the driving force of development. On the other hand, all new genomic variants must arise as exaptations, mutations occur at random, and new functions cannot be adopted without prior new structures (Tattersall 2006; 2009; 2014).

Sometimes, a combination of pre-existing elements (exaptations) results in something totally unexpected (Tattersall 2006). In the case of limb development, Clack discovered in 2005 that many of the critical innovations arose while vertebrates were still largely aquatic (Clack 2005). Furthermore, Clack suggested that the first changes appeared to have been related not strictly to locomotion but to an increased dependence on breathing air (Clack 2005).

## Promising natural model

It is believed that regular occurrence of aquatic hypoxia (low oxygen conditions), being a primary factor, led to the evolution of air breathing in the Late Silurian fishes and as a result enabled vertebrates to invade land in the Devonian period (Randall et al. 1981; Graham 1997). Also, in modern air-breathing fishes, hypoxia is the greatest inducing force for air breathing (Graham 1997; Seymour et al. 2008; Nelson 2014). Among the air-breathing fishes, the gut-breathing fishes (GBF) seem especially interesting; they must have special adaptations to use their alimentary tract as an accessory respiratory organ during low oxygen levels in the water (Nelson and Dehn 2011; Nelson 2014).

**Fig. 1:**
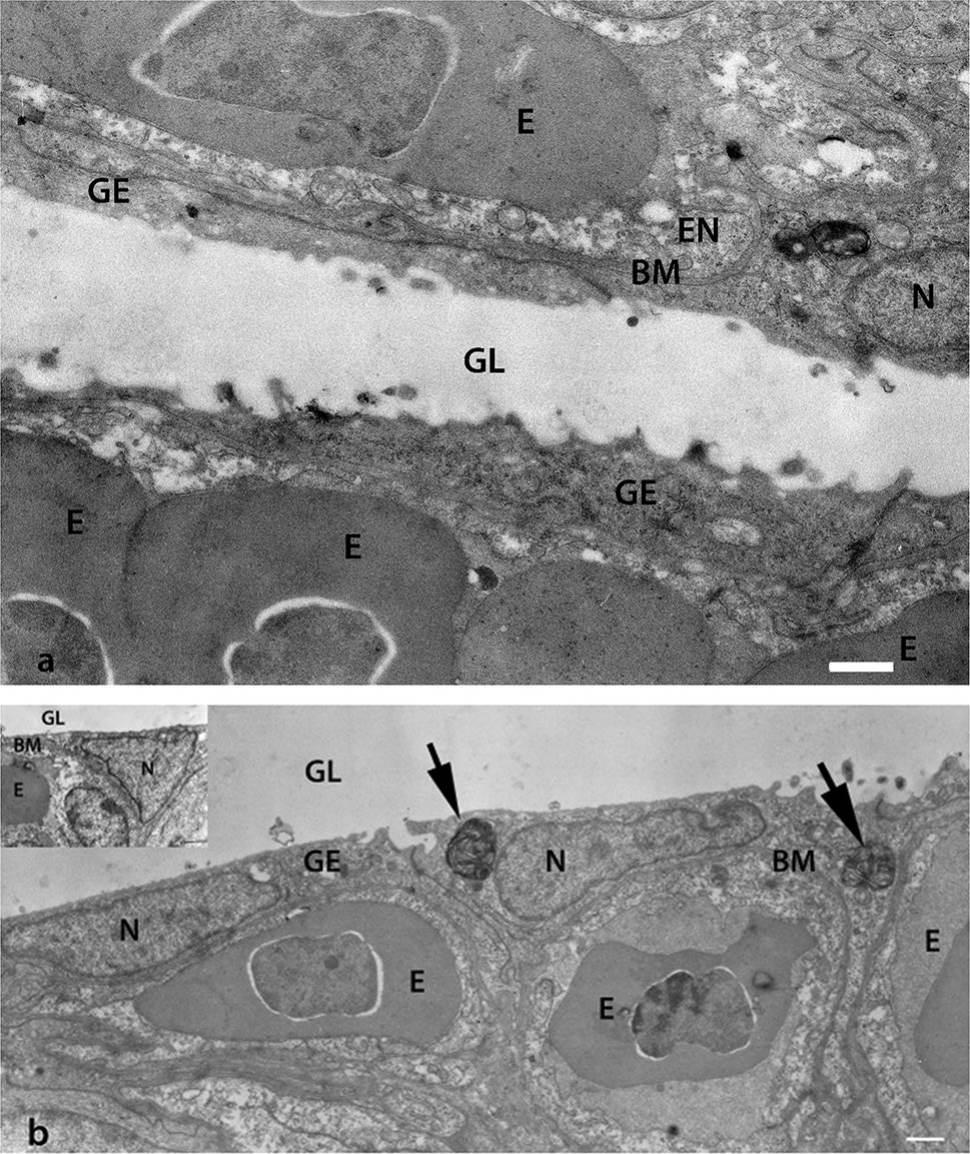
**a** Transmission electron micrograph of the gas–blood barrier in the corpus of the stomach of *Ancistrus multispinnis* (Loricariidae). The gas–blood barrier is composed of three layers: external, thin cytoplasmic sheets of respiratory epithelial cells (remodeled gastric epithelial cells), narrow interstitial space, with basement membrane (BM), and thin parts of endothelial cells (EN). *E* erythrocyte with nucleus; *GE* gastric epithelium; *GL* gastric lumen; *N* nucleus of epithelial cell. Taken from Satora (1999). Scale bar = 1 µm. **b** Transmission electron micrograph of a section of the stomach corpus epithelium of *Ancistrus multispinnis*. Flattened epithelial cells with nucleus (N) and lamellar bodies (arrows) are visible. *BM* basement membrane; *E* erythrocyte; *GE* gastric epithelium; *GL* gastric lumen. Taken from Satora (1999). Scale bar = 1 µm. The cell body with large nucleus (N) is situated between capillaries covered by thin epithelial sheets (insert in 1b). Taken from Satora (1999). Scale bar = 1 µm

**Fig. 2:**
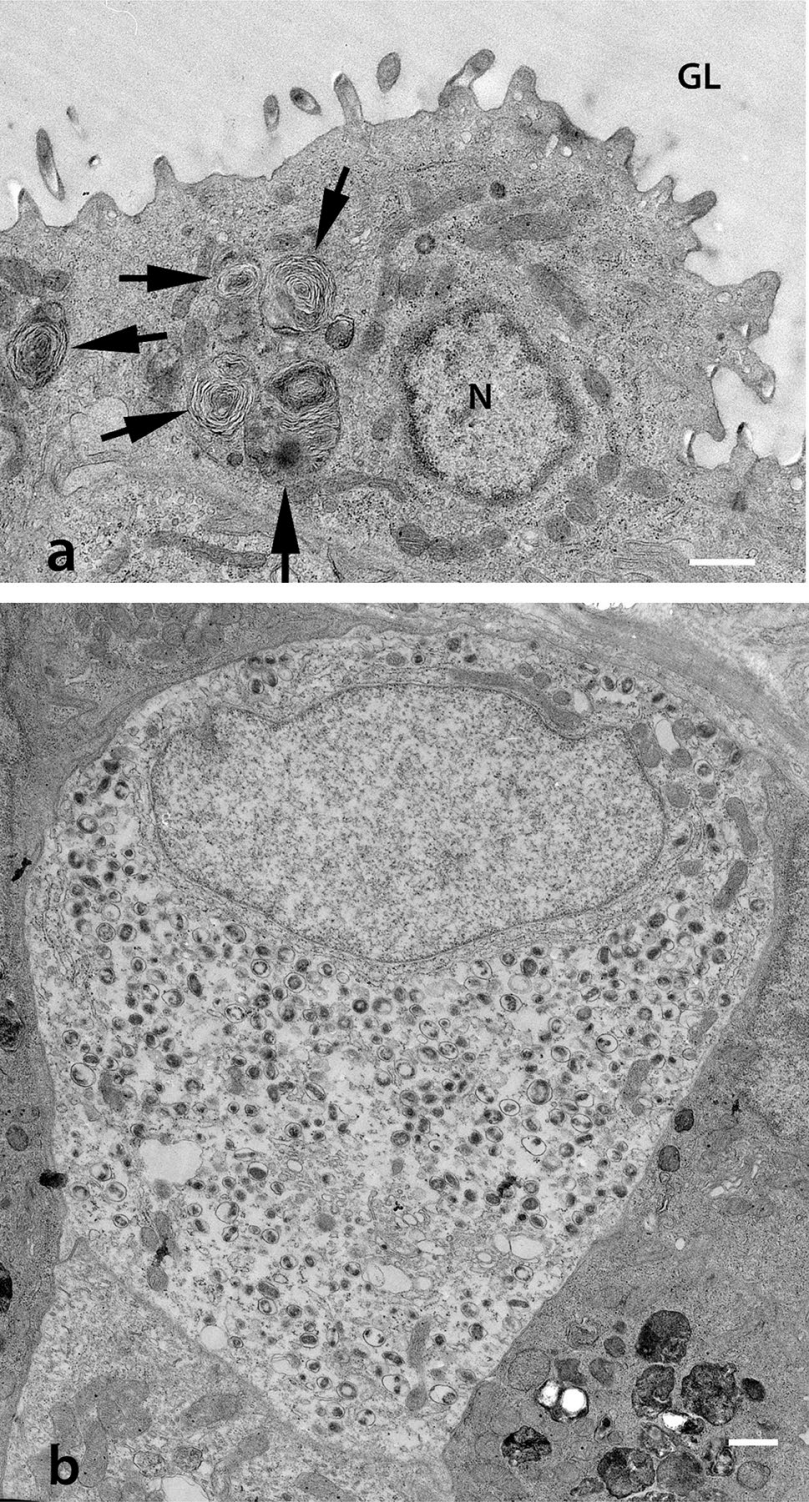
**a** Transmission electron micrograph of the stomach of *Ancistrus multispinnis* (Loricariidae). Epithelial cell with nucleus (N) and lamellar bodies (arrows). *GL* gastric lumen. Taken from Satora (1999). Scale bar = 1 µm. **b** Ultrastructure of neuroendocrine-like cell of the stomach corpus *Ancistrus multispinnis.* The cytoplasm contains characteristic secretory vesicles (dense core vesicles). Taken from Satora and Winnicki (2000). (TEM) Scale bar = 1 µm

**Fig. 3:**
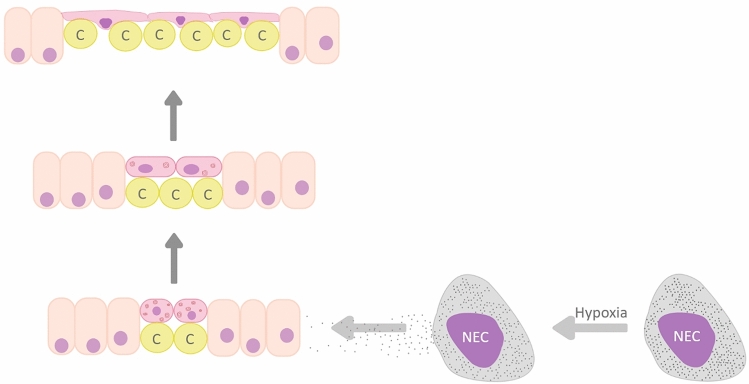
Summary schematic of “epithelial remodeling” in the gastrointestinal tract in gut-breathing fishes under hypoxic conditions. Aquatic hypoxia causes degranulation of graininess within putative chemoreceptors (NEC), which triggers a “cascade of events.” As a result, proliferation of oval epithelial cells situated between columnar enterocytes follows, combined with a change in the shape of the cells—gradual flattening and stretching. The capillaries (c) get closer in relation to the future respiratory surface. At the same time, the number of lamellar bodies contained within decreases. In the final stage of this process, the epithelial cells differentiate into the enlarged basal part (with nucleus) located between the capillary (c) and strongly flattened peripheral extensions. Numerous capillaries are covered only with flattened projections of epithelial cells, thereby creating a gas–blood barrier which enables gas diffusion

Studies on GBF showed that aquatic hypoxia induces proliferation of the squamous cells (similar to AT1 cells) in the digestive tract and makes gas diffusion possible (Fig. 1a) (Satora 1999). The phenomenon was described as a type of “epithelial remodeling” (Fig. 3) (Satora et al. 2020a, b). The majority of epithelial cells in the respiratory region of the digestive tract in the GBF are differentiated into the enlarged basal part containing the nucleus, and strongly flattened, anuclear peripheral extensions. The resulting gas–blood barrier is composed of three thin layers (Fig. 1a): capillary endothelium, basement membrane and flattened projections of epithelial cells (Jasiński 1973; Satora 1998, 1999; Podkowa and Goniakowska-Witalińska 2002, 2003; Cruz et al. 2009; Cruz and Fernandes 2016). The minimum measured thickness of this barrier is 0.2 µm (Jasiński 1973). At the same time, numerous capillary vessels are located between the bodies of the epithelial cells (Jasiński 1973; Satora 1998; Satora and Winnicki 2000). Some GBF can adjust almost 50% of the digestive tract into an additional respiratory organ under hypoxic water conditions (Leknes 2015). Also, AT2 cells of varied shape and with numerous lamellar bodies (Fig. 2a) have been observed in the GBF (Jasiński 1973; Satora 1998; Podkowa and Goniakowska-Witalińska 2002, 2003). Their role as progenitors for AT1 cells in GBF requires further research, but the observed change in their shape, which consists in flattening/extension, with the decreasing number of lamellar bodies may indicate formation of AT1 cells (Figs. 1b and 3).

Lungs, as well as respiratory and non-respiratory bladders of chondrosteans, appear to have originated from a respiratory, posterior pharynx through proliferation of the squamous cells and gradual enlargement. The fish groups which have lungs, or a pulmonoid/respiratory swim bladder, tend to develop only the skin as an accessory aerial gas exchange organ, whereas those with non-secretory or secretory swim bladder also modify their gills, opercular or branchial cavities, pharynx, pneumatic duct, stomach or intestine (Perry et al. 2019). It is suggested that this mechanism has developed independently in several species of the GBF (Satora et al. 2020b). The epidermal growth factor receptors (EGFR) seem to be among the factors responsible for the adaptation of the gastrointestinal tract to the role of additional respiratory organ in the GBF (Satora et al. 2017; Mytych et al. 2018). In mammals, the EGFR plays an important role in lung maturation; EGFR deficiency results in a mild respiratory distress syndrome and delayed lung maturation (Miettinen et al. 1997). Other essential elements include secretory neuroepithelial-like cells (NECs), putative chemoreceptors (Zaccone et al. 2017, 2018, 2019, 2020, Capillo et al. 2021; Lauriano et al. 2021), which are probably responsible for the control of proliferation of AT1 cells (Fig. 3) in the digestive tract in the GBF during hypoxia (Satora et al. 2020b).

In vertebrates, specialized sensory cell types called neuroepithelial sensors, or neuroendocrine cells (NECs), display characteristics of both neurons and hormone-secreting endocrine cells (Lauriano et al. 2021). In the mammalian lung, pulmonary neuroendocrine cells (PNEC) are widely distributed throughout the airway mucosa as solitary cells and as distinctive innervated clusters—called neuroepithelial bodies (NEB). They can detect airborne allergens and relay signals to stimulate immune cells and induce tissue/organ-wide responses. Their increase is associated with a wide range of congenital and infantile lung disorders (Cutz 2015; Jonz et al. 2016; Whitsett et al. 2019). The PNEC and NEB also play an important part in mammalian lung development (Cutz 2015; Whitsett et al. 2019). It is suggested that the groups of neuroendocrine cells represent an ancient mechanism for environmental sensing that integrates epithelial receptors with innate immunity (Lauriano et al. 2021). Understanding their role in lung regeneration and aging is of utmost importance (Cutz 2015; Branchfield et al. 2016; Sui et al. 2018; Whitsett et al. 2019). The chemoreceptors have both receptor and secretory function, and initiate reflex responses to hypoxia (Jonz et al. 2016); they were observed to be active (releasing granules) in hypoxic conditions (Tzaneva et al. 2011)—the strongest air-breathing-inducing factor (Randall et al. 1981; Graham 1997; Nelson 2014).

Additionally, in the respiratory intestine of the bronze corydoras (*Corydoras aeneus*), a hypoxia-inducible factor-1α (HIF-1α) has been found, which is considered the main transcriptional regulator of the cellular and the developmental response to hypoxia (Satora et al. 2018).

## Natural model organism

Proliferation of the squamous epithelial cells observed in the alimentary tract of the GBF in conditions of water hypoxia (Fig. 3) has probably led to the gradual evolutionary development of lungs in terrestrial vertebrates (Satora et al. 2020a). HIF-1α, depending on the normoxic/hypoxic conditions, is one of the most important downstream effector molecules of the EGFR pathway (Lu et al. 2012). Also, NECs—putative chemoreceptors (Figs. 2b,  3)—were found to play an important role in stimulating the development of organs for air breathing in the early terrestrial vertebrates (Jonz 2018; Smatresk 1990). Thus, the most important factors associated with proliferation of AT1 cells, such as HIF-1α, NECs and EGFR, are present in the GBF (Satora and Winnicki 2000; Satora et al. 2017; Mytych et al. 2018; Satora et al. 2018). GBF antibodies directed against human EGFR and HIF-1α were successfully used in immunohistochemical and western blot studies (Mytych et al. 2018; Satora et al. 2018), which additionally facilitates the observations. The research on signals and interactions between those elements in conditions of hypoxia makes it possible to observe a “switching pulse” initiating the proliferation of squamous epithelial cells (Fig. 3). In addition, the state of normoxia causes inhibition of the proliferation process. The GBF can be considered a natural research model of great potential, enabling a breakthrough in research on AT1 cell proliferation.

The presence of NECs was detected in developing sites of gas exchange in the GBF (Satora and Winnicki 2000; Podkowa and Goniakowska-Witalińska 2002, 2003). Thus, understanding the function of NECs in the formation of the squamous epithelium which enables gas diffusion in the GBF seems crucial (Satora et al. 2020b). On the other hand, experimental studies on NECs of a simple model—GBF—may lead to a breakthrough and contribute to an understanding of the processes which govern proliferation of squamous epithelium, and thus regeneration of respiratory epithelium in the lungs. Therefore, the GBF would seem to be an ideal, low-cost model organism for developmental and molecular biology, but also for physiology.

## At the end

Understanding the mechanism of proliferation of AT1 cells which enable gas diffusion is critically important. However, models using cell cultures are too simplistic and may lead to misinterpretations (Sims et al. 2005). In turn, studies using mammalian models constitute highly interactive models (Sonnenschein and Soto 2018). Thus, a relatively simple natural model which allows for easy stimulation of squamous epithelial cell proliferation is extremely valuable. Numerous experiments have shown that fishes are promising models for molecular studies, with great potential. For example, the zebrafish (*Danio rerio*) is a vertebrate model widely used in biomedical research (Bradford et al. 2017).

There is an increasing body of evidence that air breathing in tetrapods arose as an exaptation. Furthermore, the proliferative ability of squamous epithelial cells, observed in the GBF, seems to be a practical breakthrough which came into existence under the effect of environmental stimulus—hypoxia (Capillo et al. 2021). Using the GBF as a natural model organism opens a completely new avenue which is not available with other models such as mammals and cell lineages. In the studies on such models, tools dedicated to mammals (such as antibodies) can be used successfully (Mytych et al. 2018). Moreover, the model is not expensive and the experiments are relatively easy to conduct.

## In conclusion

Despite efforts, it has been impossible to identify the mechanisms which control human lung development, the precise identity and function of human lung stem and progenitor cell types, and the genetic and epigenetic control of human lung fate, without which progress toward the development of strategies for lung regeneration following injury is impossible.

The suggestion that the evolution of air breathing in all vertebrates occurred through exaptations opens a completely new research perspective. Studies on the mechanisms that control the proliferation of squamous epithelium in the alimentary canal in GBF may contribute to a precise understanding of the signal pathways which govern this process in mammals. This in turn may lead to a breakthrough in the study of mammalian lung regeneration.

## Data Availability

Not applicable.
